# Anion Effects on the Ion Exchange Process and the Deformation Property of Ionic Polymer Metal Composite Actuators

**DOI:** 10.3390/ma9060479

**Published:** 2016-06-15

**Authors:** Wataru Aoyagi, Masaki Omiya

**Affiliations:** 1 Graduate School of Science and Technology, Keio University, Yokohama, Kanagawa 223-8522, Japan; hamablue1012@outlook.jp; 2 Department of Mechanical Engineering, Keio University, Yokohama, Kanagawa 223-8522, Japan

**Keywords:** soft actuator, polymer electrolyte, anion, ionic-polymer metal composite, deformation, alternating-current impedance, 07.10.Cm, 68.60.Bs, 62.20.F-, 43.58.Bh, 77.55.-g, 82.45.Mp

## Abstract

An ionic polymer-metal composite (IPMC) actuator composed of a thin perfluorinated ionomer membrane with electrodes plated on both surfaces undergoes a large bending motion when a low electric field is applied across its thickness. Such actuators are soft, lightweight, and able to operate in solutions and thus show promise with regard to a wide range of applications, including MEMS sensors, artificial muscles, biomimetic systems, and medical devices. However, the variations induced by changing the type of anion on the device deformation properties are not well understood; therefore, the present study investigated the effects of different anions on the ion exchange process and the deformation behavior of IPMC actuators with palladium electrodes. Ion exchange was carried out in solutions incorporating various anions and the actuator tip displacement in deionized water was subsequently measured while applying a step voltage. In the step voltage response measurements, larger anions such as nitrate or sulfate led to a more pronounced tip displacement compared to that obtained with smaller anions such as hydroxide or chloride. In AC impedance measurements, larger anions generated greater ion conductivity and a larger double-layer capacitance at the cathode. Based on these mechanical and electrochemical measurements, it is concluded that the presence of larger anions in the ion exchange solution induces a greater degree of double-layer capacitance at the cathode and results in enhanced tip deformation of the IPMC actuators.

## 1. Introduction

An ionic polymer-metal composite (IPMC) consists of a thin perfluorinated ionomer membrane with electrodes plated on both faces. When the membrane is hydrated, a device of this type will undergo a large bending motion as a low level electric field is applied across the two electrodes [1,2,3,4]. When preparing an IPMC actuator, the device is first immersed in a solution containing cations, and hydrogen ions in the ionomer are replaced with the cations. On the subsequent application of a voltage of approximately 1 to 3 V to the ion-exchanged IPMC, the adsorbed cations will migrate to the cathode side; consequently, the volume of the cathode side will increase while the volume of the anode side decreases. This effect causes the actuator to bend toward the anode side [5,6,7,8]. IPMC actuators can be readily miniaturized and typically have a low density and high mechanical flexibility, such that they show promise for a wide variety of applications, ranging from MEMS sensors to artificial muscles [9,10,11,12,13,14,15,16,17,18,19].

The mechanical and electrochemical characteristics of IPMC actuators have been studied by many researchers [20,21,22,23,24,25,26,27,28,29]. However, the effects of environmental conditions on the deformation properties of these devices remain unclear [30]. In particular, there have been very few studies of the effects of anions. Lee *et al.* [31] found that the particular anion associated with imidazolium ionic liquids affects the deformation behavior of IPMC actuators, and also identified effects based on the size and ionic mobility of the anion of the imidazolium salt. In the case of IPMC actuators based on aqueous solutions, Nafion™ membranes (Dupont, Wilmington, DE, USA) cannot be readily doped with anions due to the negatively charged R-SO_3_^−^ groups in the membrane. However, it is still possible that anions may affect the ion exchange process of cations; thus it is very important to clarify the effect of various anions on the ion exchange process and the deformation properties of these devices when developing systems that employ IPMC actuators [32]. For larger deformation, applying a high voltage is one method. However, when high voltage is applied, the efficiency of actuator becomes worse due to the electrolysis of water and the joule heating on electrodes. Therefore, it is necessary to use IPMC actuators under low applied voltage.

This study investigated the deformation mechanism of IPMC actuators when combined with four different types of anions, and using a palladium electrode [33], since palladium is less expensive than conventional electrode materials such as gold or platinum. The deformation behavior of the actuators before and after ion exchange was observed in deionized water. Additionally, alternating current (AC) impedance measurements were conducted on the actuators following cation exchange under four different types of anions in order to measure the bulk resistance of the Nafion™ membrane as well as the charge transfer resistance and double-layer capacitance at the electrode/membrane interfaces associated with both the cathode and anode. Based on the results of the AC impedance measurements, we discuss the effect of anions on the ion exchange process and the deformation properties of IPMC actuators with palladium electrodes.

## 2. Experimental Methods

### 2.1. Preparation of IPMC Actuators

**Figure 1:**
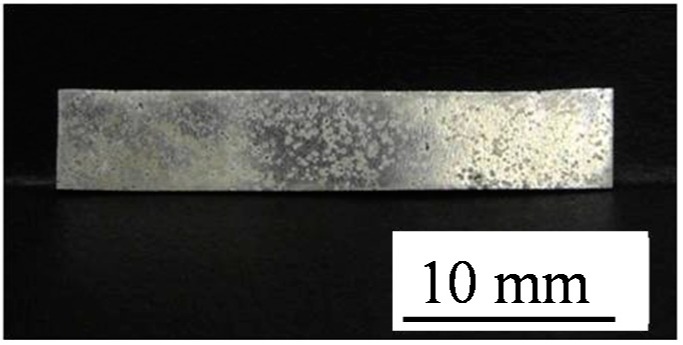
Ionic polymer-metal composite (IPMC) actuator specimen.

Twelve IPMC actuators with palladium electrodes were prepared by non-electrolytic plating [33]. A Nafion 117™ membrane (Dupont, Wilmington, DE, USA) was used as the ion conductive polymer since this material is chemically inert, thermally stable, and has excellent electrochemical properties [34]. After forming palladium electrodes on the membrane, an IPMC actuator was fabricated by cutting the plated membrane into sections with dimensions of 28 mm × 5 mm, such that the electrodes on both faces were isolated from one another. Figure 1 shows an image of one such IPMC actuator sample. These actuators were stored in deionized water prior to use in experiments.

### 2.2. Evaluation of Actuator Response to a Step Voltage

**Figure 2:**
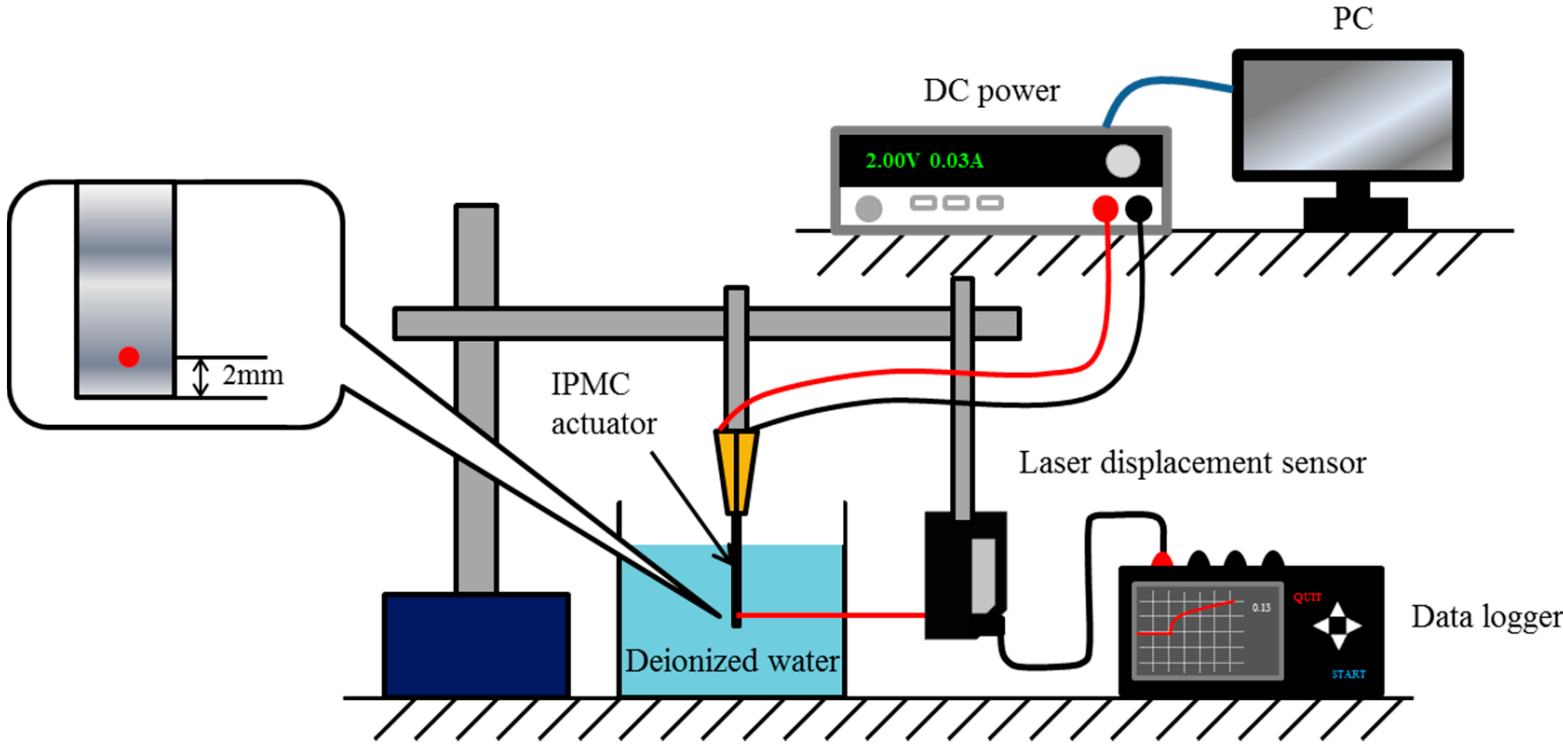
Experimental setup for evaluating the response to step voltage.

To evaluate the anion effect on the deformation behavior during the application of a step voltage, two measurements were conducted. Figure 2 shows a schematic diagram of the experimental setup used to evaluate the actuator response to the step voltage. An IPMC actuator (length: 28 mm; effective length: 25 mm; width: 5 mm) was placed in a container filled with deionized water. A laser displacement sensor (Keyence, LB-040, Osaka, Japan) was then set beside the container, and the displacement was measured at a position 2 mm from the bottom of the actuator. A DC power supply (Agilent, E3640A, Santa Clara, CA, USA) was used to apply a 2.0-V step voltage for 20 s to the actuator while in deionized water, and the tip displacement measured by the laser displacement sensor was logged by a digital recorder (Graphtec, midi LOGGER GL900, Yokohama, Japan). In addition, the current was recorded using the LabVIEW (National Instruments, Austin, TX, USA) software.

#### 2.2.1. Experiment 1: Responses to a Step Voltage before Ion Exchange

Step voltages of 2.0 V were applied to 12 IPMC actuator specimens that had been stored in deionized water after fabrication, and the resulting tip displacement and current for each specimen were recorded. Measurements were conducted three times for each specimen.

#### 2.2.2. Experiment 2: Responses to a Step Voltage after Ion Exchange

Following the above trials, the same 12 specimens were divided into four groups of three specimens, and were immersed in four different aqueous solutions (as shown in Table 1) for one day to allow ion exchange to take place. It should be noted that all four solutions had the same sodium ion concentration (0.1 mol/L). Following the ion exchange process, the same measurements as had been performed in the initial experimental series were conducted.

### 2.3. Alternating Current Impedance Measurements

The AC impedance of each sample was measured via the conventional two-probe method, using a potentiostat/galvanostat (Princeton Applied Research, VersaStat 4, Oak Ridge, TN, USA). The measurement frequency range was from 1 to 0.1 MHz, using an input AC voltage of 10 mV. Four IPMC actuators were selected for these measurements, one from each of the different anion groups.

#### 2.3.1. Experiment 3: AC Impedance Measurements before Ion Exchange

Prior to the Experiment 1 trials, AC impedance measurements were performed three times for each of the four IPMC actuators noted above.

#### 2.3.2. Experiment 4: AC Impedance Measurements after Ion Exchange

Following the impedance measurements associated with the Experiment 3 trials, the four IMPCs noted in Section 2.2.2 were subjected to ion exchange, after which the same impedance measurements were repeated prior to the Experiment 2 trials.

## 3. Experimental Results

### 3.1. Deformation Behavior in Experiments 1 and 2

**Figure 3:**
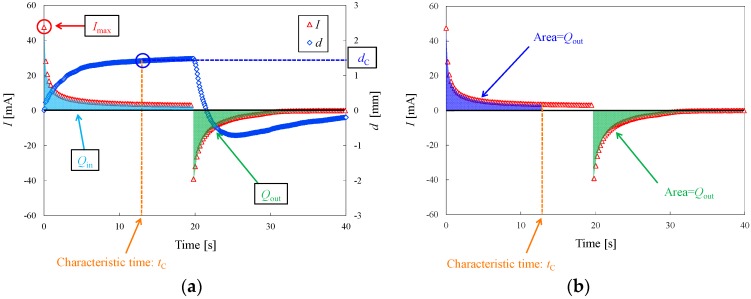
Actuator response to step voltage in deionized water and the definition of evaluation parameters. (**a**) Definitions of *I*_max_, *d*_C_, *Q*_in_, and *Q*_out_; (**b**) Definition of characteristic time.

Figure 3 shows typical responses to step voltage obtained during the Experiment 1 trials, while Figure 3a,b presents the definitions of the parameters used for evaluation of the deformation properties of the actuators. Four parameters, *I*_max_, *d*_C_, *Q*_in_, and *Q*_out_, were used for evaluation purposes. The maximum current, *I*_max_, represents the maximum current measured during the application of voltage. The charge intensity, *Q*_in_, is the total quantity of electrons that flow to the cathode, and is given by the area indicated in Figure 3a. The discharge intensity, *Q*_out_, represents the total quantity of electrons that flow from the cathode, and is equal to the area indicated in Figure 3a. The characteristic displacement, *d*_C_, is the displacement at the characteristic time, *t*_C_, defined as the time at which the amount of the electrons flowing to the cathode equals the discharge intensity, *Q*_out_, shown in Figure 3b.

**Figure 4:**
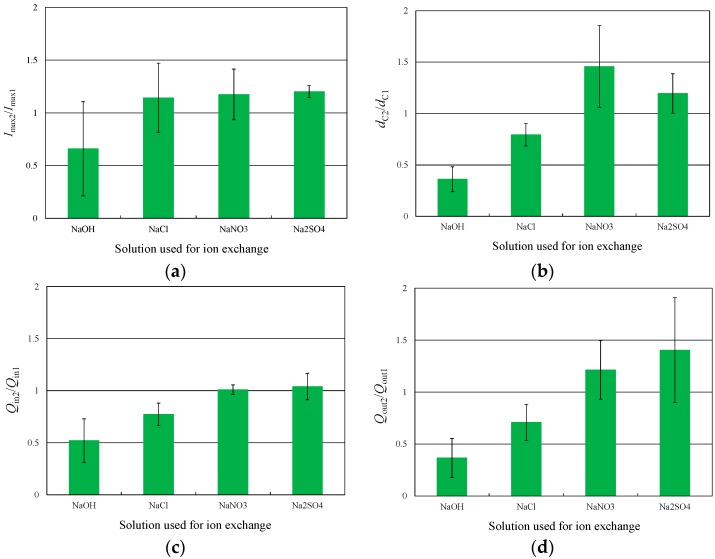
(**a**) Relationship between maximum current, *I*_max_, and anion types; (**b**) Relationship between characteristic displacement, *d*_C_, and anion types; (**c**) Relationship between charge intensity, *Q*_in_, and anion types; (**d**) Relationship between discharge intensity, *Q*_out_, and anion types.

The above four parameters were calculated from the results of Experiments 1 and 2, and the ratio of the value of each parameter before and after ion exchange was calculated. Figure 4a–d presents the average values thus obtained, along with the associated standard deviations. Here, subscript 1 indicates the results of Experiment 1 and subscript 2 refers to Experiment 2. The characteristic displacement, *d*_C_, and discharge intensity, *Q*_out_, both increased after ion exchange in the NaNO_3_ and Na_2_SO_4_ solutions. In contrast, *d*_C_ and *Q*_out_ both decreased after ion exchange in the NaOH and NaCl solutions. The charge intensity, *Q*_in_, also decreased after ion exchange in the NaOH and NaCl solutions. These results confirm that the particular anion present in the aqueous solution used for ion exchange affects the deformation behavior of the IPMC actuator.

### 3.2. Alternating-Current Impedance Measurement

**Figure 5:**
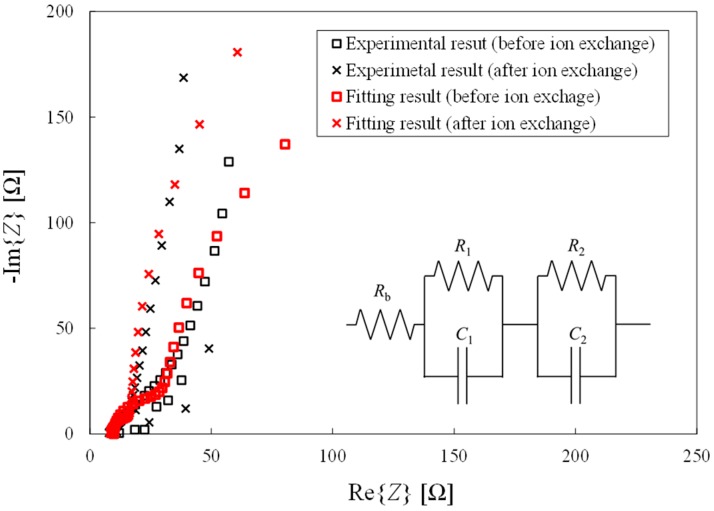
Comparison of experimental and fitting results for Nyquist plots before and after ion exchange in 0.1 mol/L NaCl aq.

Figure 5 shows Nyquist plots obtained for an IPMC in deionized water before and after ion exchange in an aqueous NaCl solution. The equivalent circuit pictured in Figure 5 was used for curve fitting to the experimental results, using impedance analysis software (Princeton Applied Research, Z view, Oak Ridge, TN, USA). The equivalent circuit was determined based on publications by Paquette *et al.* [35] and Ma *et al.* [36]. Here, *R*_b_ is the bulk resistance of the Nafion™ membrane, *R*_1_ and *R*_2_ are the charge transfer resistances at the two electrode/membrane interfaces, and *C*_1_ and *C*_2_ are the double-layer capacitance values at the two electrode/membrane interfaces, respectively. The parallel circuit consisting of *R*_1_ and *C*_1_ represents the anode, while the parallel circuit consisting of *R*_2_ and *C*_2_ represents the cathode.

**Figure 6:**
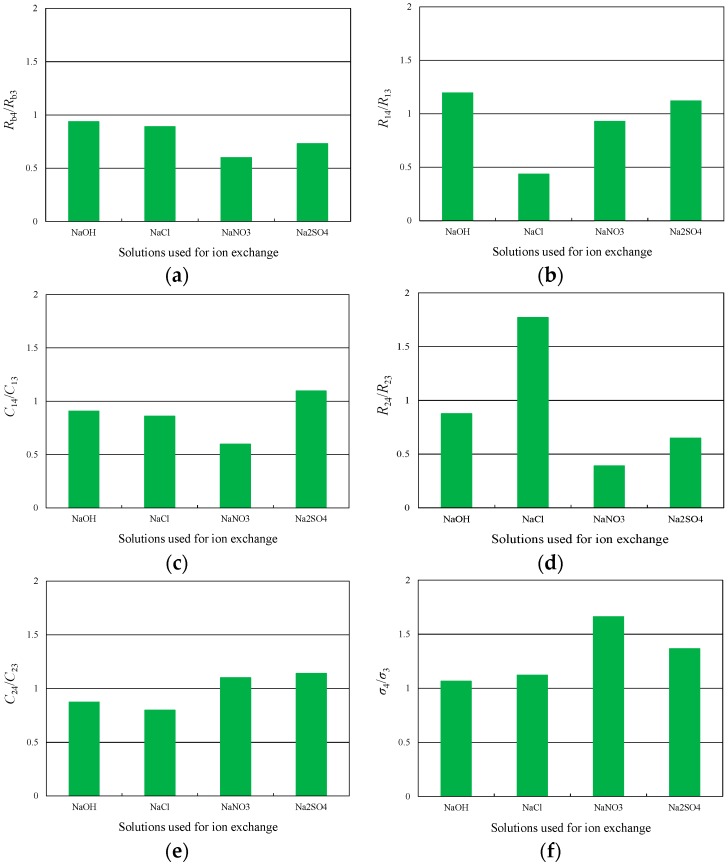
(**a**) Relationship between bulk resistance, *R*_b_, and anion types; (**b**) Relationship between charge transfer resistance at the anode, *R*_1_, and anion types; (**c**) Relationship between double-layer capacitance at the anode, *C*_1_, and anion types; (**d**) Relationship between charge transfer resistance at the cathode, *R*_2_, and anion types; (**e**) Relationship between double-layer capacitance at the cathode, *C*_2_, and anion types; (**f**) Relationship between ion conductivity, *σ*, and anion types.

The values of five parameters, *R*_b_, *R*_1_, *R*_2_, *C*_1_, and *C*_2_, were calculated by this curve fitting approach, based on the equivalent circuit model. The associated ion conductivity, *σ*, was also calculated from

(1) $$\sigma =\frac{b}{R_{b}A}$$

where *b* is the thickness of the Nafion™ membrane, and *A* is the electrode surface area. The plots in Figure 5 indicate a degree of scatter in the data obtained at higher frequencies (*f* > 1000 Hz) and a resonant frequency of this size is less than 10 Hz. Therefore, curve fitting was performed without the high frequency data. From Figure 5, it is confirmed that this equivalent circuit can properly model the behavior of the actuator. The ratios of each of the above parameters before and after ion exchange were also calculated, and the average results are summarized in Figure 6, in which subscripts 3 and 4 refer to the results of Experiments 3 and 4, respectively. Following ion exchange in the NaNO_3_ and Na_2_SO_4_ solutions, the charge transfer resistance at the cathode, *C*_2_, increased, while the ion conductivity, σ, was found to greatly increase. Conversely, *C*_2_ decreased and σ somewhat increased following ion exchange in the NaOH and NaCl solutions.

## 4. Discussion

### 4.1. Correlation between Characteristic Displacement and Other Parameters

In order to identify the parameters that are closely connected to the characteristic displacement, the correlation coefficient, *COR*, for the relationship between the characteristic displacement and other parameters was evaluated based on the ratio of the values obtained before and after ion exchange; the resulting *COR* values are listed in Table 2. There are evidently strong positive correlations between the characteristic displacement, *d*_C_, and the charge intensity, *Q*_in_, the discharge intensity, *Q*_out_, and the ion conductivity, *σ*. Table 2 also shows positive correlations between *d*_C_ and the double-layer capacitance at the cathode, *C*_2_. Therefore, the following discussion focuses on the relationship between the anion type and *d*_C_, *Q*_in_, *Q*_out_, *σ*, and *C*_2_.

### 4.2. Relationship between Ion Conductivity and Ion Exchange

The structure of the Nafion™ membrane was analyzed by Gierke [37], who found that the membrane consists of separate hydrophilic and hydrophobic regions. In this material, spherical hydrophilic clusters exist in a hydrophobic matrix, connected by hydrophilic channels about 1 nm in diameter. It is believed that ions and water molecules migrate through these channels.

After ion exchange, the ion conductivities of all IPMCs were found to have increased (see *σ*_4_/*σ*_3_ in Table 2). This occurs because sodium ions take the central role in ion conduction following substitution for hydrogen ions during the ion exchange process. Hydronium and sodium ions have been reported to have hydration numbers of 4.5 and 3.9, respectively [38]; thus, protons have more associated water of hydration and are consequently larger than sodium ions when hydrated in solution. It is therefore more difficult for hydrogen ions to pass through the channels in the membrane since they collide with channel walls or other hydrated cations. Therefore, ion exchange leads to an increase of ion conductivity.

### 4.3. Relationship between Parameters at the Cathode and Ion Exchange

Following ion exchange in the sodium chloride solution, the charge transfer resistance at the cathode was remarkably increased (see *R*_24_/*R*_23_ in Table 2). In addition, the double-layer capacitance at the cathode became smaller after ion exchange in the sodium hydroxide and sodium chloride solutions, but increased following treatment in the sodium nitrate and sodium sulfate solutions (see *C*_24_/*C*_23_ in Table 2). These two results indicate that the concentration of sodium ions in the membrane may be different depending on the types of anions during the ion exchange process, even though the concentration of sodium ions was controlled to be the same in ion exchange solutions.

The sodium ion exchange process in the cation exchange membrane is explained by a Donnan membrane equilibrium equation [39] and the concentration of sodium ion, ${\bar{C}}_{{\text{Na}}^{+}}$, and anion, ${\bar{C}}_{X}$, in the membrane can be approximately calculated as (see Appendix)

(2) $${\bar{C}}_{{\text{Na}}^{+}}=\frac{1}{2}\left\{\sqrt{{\bar{C}}_{R}^{2}+4f_{{\text{Na}}^{+}}f_{X}C_{{\text{Na}}^{+}}C_{X}}+{\bar{C}}_{R}\right\}$$

(3) $${\bar{C}}_{X}=\frac{1}{2}\left\{\sqrt{{\bar{C}}_{R}^{2}+4f_{{\text{Na}}^{+}}f_{X}C_{{\text{Na}}^{+}}C_{X}}-{\bar{C}}_{R}\right\}$$

where $C_{{\text{Na}}^{+}}$ is the concentration of sodium ion in solution, $C_{X}$ the concentration of anion *X* in solution, and $f_{{\text{Na}}^{+}}$, $f_{X}$ the activity coefficient of sodium ion and anion *X*, respectively. From Equation (2), it is noted that the concentration of Na^+^ in the membrane depends on the concentration of Na*^+^* and anion *X* in the solution, the concentration of cation exchange group in the membrane, and the activity coefficient of cation and anion in the solution.

The activity coefficient of ion is approximately expressed by Debye–Hückel equation [40],

(4) $$\log f_{i}=-\frac{Az_{i}^{2}\sqrt{I}}{1+\beta a\sqrt{I}}$$

where *A* is the Debye-Hückel constant, and *I* the ionic strength. *βa* is a constant related to hydrated ionic radius, which is unity when *a* = 3.04 Å. From Equations (2) and (4), it is noted that larger hydrated ionic radius induces larger activity coefficient. The order of hydrated anion radius is OH^−^ < Cl^−^ < NO_3_^−^ < SO_4_^2−^ [41,42]. This indicates that the concentration of the sodium ion in the membrane is one reason for different electrochemical properties between ion exchange solutions.

### 4.4. The Relationship between Characteristic Displacement and Ion Exchange

As described in Section 3.1, the characteristic displacement, *d*_C_, and the discharge intensity, *Q*_out_, increased after ion exchange in the NaNO_3_ and Na_2_SO_4_ solutions, while both decreased after ion exchange in the NaOH and NaCl solutions. The charge intensity, *Q*_in_, also decreased after ion exchange in NaOH and NaCl solutions. These results are explained as follows.

When ion exchange was performed in the solutions containing small anions, the concentration of sodium ion in the membrane had a tendency to decrease compared to the concentration obtained with the use of larger anions. The resistance associated with hydrated cations migration also increased under these conditions compared to the use of larger anions, and the extent to which ion conductivity was improved by ion exchange was decreased, as was the double-layer capacitance at the cathode. It can be considered that these changes following ion exchange result in a decreased charge and discharge intensity, and in a decrease in the characteristic displacement on the application of voltage.

In contrast, when ion exchange was performed in the solutions containing large anions, the concentration of sodium ion in the membrane tended to increase to a greater extent compared with that obtained in the case of ion exchange by small anions. Under these conditions, the resistance associated with hydrated cations migration was reduced, and the extent to which the rate of ion conductivity increased following ion exchange was enhanced, as was the double-layer capacitance at the cathode. It appears that these changes after ion exchange result in an increase in the discharge intensity, although the charge intensity does not change, and this is the cause of the increase in the characteristic displacement after ion exchange in NaNO_3_ and Na_2_SO_4_ solutions.

## 5. Conclusions

The mechanical and electrochemical properties of IPMC actuators with palladium electrodes were evaluated before and after ion exchange in various aqueous solutions containing different anions. The response to a 2-V step voltage was measured to examine their deformation properties, and impedance measurements were performed to evaluate the mobility of sodium ions in the IPMCs. Based on the results of these two measurements and the above discussion, we conclude the following:

1. In the response to 2-V step voltages, the use of smaller anions decreases the charge intensity, discharge intensity, and the characteristic displacement of the IPMC actuator. In contrast, larger anions increase the discharge intensity and characteristic displacement.
2. The AC impedance measurement results indicate that smaller anions increase the charge transfer resistance at the cathode and decrease the double-layer capacitance at the cathode. Conversely, larger anions decrease the charge transfer resistance and increase the double-layer capacitance. Furthermore, the ion conductivity greatly increases after ion exchange in NaOH and NaCl solutions.
3. The main effect of anion sizes is appeared in the ion exchange process. Anions hardly migrate into the Nafion membrane which has the negatively charged R-SO^3−^ groups. Therefore, anions do not directly affect the deformation principle of IPMC actuators. We consider that anion sizes affect the cation exchange process and that the cation concentration in the membrane directly affects the deformation principle of IPMC actuators.
4. When ion exchange was performed in solutions containing larger anions, the cation concentration in the membrane tended to increase. The resistance to hydrated cations migration was reduced under these conditions; thus, the extent to which ion conductivity was increased by ion exchange became large, as did the double-layer capacitance at the cathode. These changes following the ion exchange with solutions incorporating larger anions increased the tip displacement of the IPMCs.

## Figures and Tables

**Table 1:** Aqueous solutions used for ion exchange.

Solution	NaOH aq	NaCl aq	NaNO_3_ aq	Na_2_SO_4_ aq
Concentration (mol/L)	0.1	0.1	0.1	0.05

**Table 2:** Correlation between characteristic displacement and other parameters.

Parameters	NaOH	NaCl	NaNO_3_	Na_2_SO_4_	*COR*
*d*_C2_/*d*_C1_	0.361	0.794	1.458	1.196	–
*Q*_in2_/*Q*_in1_	0.521	0.773	1.009	1.040	0.961
*Q*_out2_/*O*_out1_	0.366	0.710	1.214	1.405	0.921
*σ*_4_/*σ*_3_	1.065	1.123	1.664	1.365	0.935
*R*_14_/*R*_13_	1.197	0.437	0.930	1.122	−0.067
*C*_14_/*C*_13_	0.908	0.861	0.599	1.098	−0.348
*R*_24_/*R*_23_	0.878	1.772	0.391	0.649	−0.513
*C*_24_/*C*_23_	0.873	0.799	1.101	1.142	0.795

## Appendix

The sodium ion exchange process in cation exchange membrane is explained by a Donnan membrane equilibrium equation as [39]

(A1) $${\bar{a}}_{{\text{Na}}^{+}}^{\nu_{{\text{Na}}^{+}}}{\bar{a}}_{X}^{\nu_{X}}=a_{{\text{Na}}^{+}}^{\nu_{{\text{Na}}^{+}}}a_{X}^{\nu_{X}}$$

where ${\bar{a}}_{{\text{Na}}^{+}}$ is the activity of sodium ion in membrane, ${\bar{a}}_{X}$ the activity of anion X in membrane, $a_{{\text{Na}}^{+}}$ is the activity of sodium ion in solution phase, $a_{X}$ is the activity of anion X in solution phase. $\nu_{{\text{Na}}^{+}}$, and $\nu_{X}$ are the number of moles of Na^+^ and anion *X*, respectively.

Considering the activity coefficient of ion, *f*, in the solution phase, the distribution of Na^+^ and *X* between the membrane and solution for monovalent ions is approximately expressed as follows:

(A2) $${\bar{C}}_{{\text{Na}}^{+}}\times {\bar{C}}_{X}=f_{{\text{Na}}^{+}}C_{{\text{Na}}^{+}}\times f_{X}C_{X}$$

where $\nu_{{\text{Na}}^{+}}=\nu_{X}$. From the electroneutrality condition in the membrane,

(A3) $${\bar{C}}_{{\text{Na}}^{+}}={\bar{C}}_{X}+{\bar{C}}_{R}$$

where ${\bar{C}}_{R}$ is the concentration of cation exchange group (R-SO_3_^−^) in the membrane. The concentration of Na^+^ and anion X in the membrane can be approximately calculated from Equations (A2) and (A3):

(A4) $${\bar{C}}_{{\text{Na}}^{+}}=\frac{1}{2}\left\{\sqrt{{\bar{C}}_{R}^{2}+4f_{{\text{Na}}^{+}}f_{X}C_{{\text{Na}}^{+}}C_{X}}+{\bar{C}}_{R}\right\}$$

(A5) $${\bar{C}}_{X}=\frac{1}{2}\left\{\sqrt{{\bar{C}}_{R}^{2}+4f_{{\text{Na}}^{+}}f_{X}C_{{\text{Na}}^{+}}C_{X}}-{\bar{C}}_{R}\right\}$$

For divalent ions such as Na_2_SO_4_, the distribution of Na^+^ and X between the membrane and solution is approximately expressed as follows:

(A6) $${\bar{C}}_{{\text{Na}}^{+}}{}^{2}\times {\bar{C}}_{X}={\left(f_{{\text{Na}}^{+}}C_{{\text{Na}}^{+}}\right)}^{2}\times \left(f_{X}C_{X}\right)$$

From electroneutrality condition in the membrane,

(A7) $${\bar{C}}_{{\text{Na}}^{+}}=2{\bar{C}}_{X}+{\bar{C}}_{R}$$

where ${\bar{C}}_{R}$ is the concentration of cation exchange group (R-SO_3_^−^) in the membrane. The concentration of Na^+^ in the membrane can be approximately calculated by solving the following equation:

(A8) $${\bar{C}}_{{\text{Na}}^{+}}{}^{3}-{\bar{C}}_{R}\times {\bar{C}}_{{\text{Na}}^{+}}{}^{2}-2{\left(f_{{\text{Na}}^{+}}C_{{\text{Na}}^{+}}\right)}^{2}\times \left(f_{X}C_{X}\right)=0$$

Qualitatively, if *f_X_* increases, the concentration of sodium ion in the membrane tends to increase. Therefore, the hydrated radius of the anion is an important parameter for the concentration of sodium ion in the membrane.
